# Annotations

**Published:** 1911-07-22

**Authors:** 


### ANNOTATIONS.

### Deaths after Eating.

The death of a convict aged thirty-six, after a meal of lettuce, bread-and-butter, and cocoa, draws attention to the fact that every year a considerable number of people die in whom after death no cause for death can be found. In this particular case the brief notice in the daily paper does not mention what symptoms were present during life. It is simply stated that the doctors who made the post-mortem examination were unable to state the cause of death. Although deaths due to food-poisoning are generally preceded by symptoms referable to disturbance of the alimentary canal, a certain number of cases are met with in the post-mortem room in which rapid death has occurred, but where such symptoms as vomiting or diarrhœa have been absent. Isolated cases of this character, as a rule, cannot be definitely traced to any article of diet, yet, in the absence of any discoverable cause of death, it is most reasonable to suppose that the fatal event has resulted from the entrance of some virulent micro-organism associated with food. How many deaths there may be of this nature throughout the country during the year is perhaps scarcely realised. Where there is no post-mortem examination the medical attendant, though probably puzzled, fills in the death certificate according to his own conviction. The experience of the post-mortem room, however, shows that were examinations after death universal, although these cases of death of obscure nature cannot be described as numerous, there must be a considerable number every year throughout the country.

### Morbid Hypersensitiveness.

The evidence given regarding the mental condition of the young man who attacked the motor cyclist is a striking instance of the pathos in the life of some products of our boasted civilisation. An autobiography was not long ago written by some one who described the life of the morbidly shy as a continual crucifixion. Self-consciousness is said to be one of the main mental characteristics which raise us above the level of the lower animals. Our self-respect and the actions which lead us to endeavour to maintain our self-respect are, needless to say, inseparable from self-consciousness. The very absence (so-called) of self-consciousness may be due to a calm confidence in the importance of personality. When, however, this confidence in personality is weakened or destroyed, every fellow human being becomes in some degree a terror, and may be magnified into an enemy. Curiously enough the morbidly self-conscious may be endowed with great gifts which under the influence of personal ambition aided by strength of will may raise the hypersensitive man to a high position, as was the case with an influential French Minister of comparatively recent years, who was said to be so nervous when interviewed that to control his feelings he was obliged to be continually screwing up his coat-tails. The hypersensitive, however, endowed with fewer gifts and perhaps devoid of personal attraction, will in unsympathetic surroundings either develop into a being utterly crushed and walk this earth in a hopeless longing for sympathy, or will become something of an Ishmael with "his hand against every man and every man's hand against him."

### The Royal Commission on Tuberculosis.

Opportunely, in view of prospective legislation, comes the publication of the Report of the Royal Commission on Tuberculosis. The labours of the Commission have extended over a period of ten years, during which a vast amount of evidence has been sifted and very valuable interim reports have been issued. It seems a pity that the complicated machinery which has been set running smoothly and usefully should now be taken to pieces instead of being kept in working order; there are still many problems and details requiring further elucidation which could profitably employ the trained workers, and for which the accumulated store of experimental records would prove of perpetual value. The final report of this Commission has the very great merit of being unanimous, and should decidedly strengthen the hands of the promoters of legislation to secure purer milk and to eliminate tuberculosis among cattle. These aims will necessitate the expenditure of large sums of money, and the public will naturally insist on a strong case being brought forward. This in itself is a justification for the continued existence of much of the machinery of the present Commission in order that it may add further to the authoritative literature and experimental evidence on this involved subject. Up to the present the work of the Commission demonstrates that "a considerable amount of the tuberculosis in childhood is to be ascribed to infection with bacilli of the bovine type transmitted to children in meals consisting largely of the milk of the cow." In adults and adolescents the bovine type of bacillus was found only rarely, but it is suggested that among the tuberculous population generally bovine bacilli are probably much more frequently present, and are responsible for a good deal of incapacity and ill-health. While pulmonary tuberculosis was found in adults to be very rarely associated with the bovine bacillus, in the primary abdominal tuberculosis of young children this type of bacillus was present in nearly half the fatal cases, and also in a large proportion of cases of cervical gland tuberculosis. The Commissioners urge more stringent regulation and supervision of milk production and of the preparation of meat, and repeat their observations regarding the danger arising from the consumption of infected milk. In their third interim report it was pointed out that not only may tubercle bacilli be present in the milk of cows presenting no obvious disease of the udder, but that the milk of tuberculous cows, free from bacilli as it leaves the udder, may and often does become infected by being contaminated by fæcal or uterine discharges of such a diseased animal.

